# Comparison of CD4+ and CD8+ T- lymphocyte in Helicobacter pylori-negative functional dyspepsia

**DOI:** 10.22088/cjim.11.2.150

**Published:** 2020

**Authors:** Kourosh Masnadi-Shirazi, Seyed Kazem Mirinezhad, Shabnam Salehi, Monireh Halimi, Leila Vahedi, Sousan Mir najd Gerami

**Affiliations:** 1 Liver and Gastrointestinal Diseases Research Center, Imam Reza Hospital, Tabriz University of Medical Sciences, Tabriz, Iran; 2 Department of Pathology, Imam Reza Hospital, Tabriz University of Medical Sciences, Tabriz, Iran

**Keywords:** Functional dyspepsia, Comparison, T-lymphocytes, Helicobacter pylori, CD4, CD8

## Abstract

**Background:**

Functional dyspepsia (FD) is the most common gastrointestinal disorder with several symptoms such as stomach pain and abdominal bloating. The aim of this study was to investigate and compare CD4+ and CD8+ in the Helicobacter pylori-negative functional dyspepsia and control groups.

**Methods:**

Sixty one patients (35 patients with stomach pain and 26 with abdominal bloating), and 30 controls were reviewed based on the quantity of CD4+ and CD8+ T-cells isolated from gastric mucosa biopsy samples. The comparison between variables was analyzed with a chi-square or Fisher’s exact test and logistic regression analyses. P<0.05 and odds ratio (OR) with a 95% confidence interval demonstrated statistical significance.

**Results:**

A significant difference was observed between two-group patients and control group based on CD4+ and CD8+ presence, respectively (P=0.003, P=0.008). Furthermore, there was a significant difference between stomach pain-patients and control group with regard to CD4 count (P=0.01) and between abdominal bloating-patients and control group with regard to CD8 count (P=0.002). There was a decrease in both CD4+ and CD8+ T-cells in gastric mucosa in patients with FD with a significant reduction in the stomach pain-patients and abdominal bloating-patients in the number of CD4+ and CD8+ T-cells, respectively.

**Conclusion:**

These results indicated that the role of immunology in the absence of the CD4+ and CD8+ T-cells in the gastric mucosa may have a protective role against FD.

Functional dyspepsia (FD) is one of the most common functional gastrointestinal disorders with a high prevalence throughout the world (1-2). The global prevalence of FD ranges from 11.7% in Asia, 20.6% in Europe, to 29% in the US and 66.6% in Africa (3, 4). FD is usually characterized by abdominal discomfort or pain with no obvious cause that could be identified by conventional diagnostic means like endoscopy (5, 6). Although the exact pathophysiology of FD remains unclear, researches indicate that a number of factors may play a role in the development of symptoms (5-7). The increasing perception of distention, impaired or altered perception of acid, visceral hypersensitivity secondary to chronic inflammation, reduced relaxation of the gastric fundus, decreased or impaired gastric emptying, changes of the gastric electric rhythm, gastroesophageal reflux and duodena-gastric reflux in the patient lead to dyspepsia. Different factors such as changes in acid secretion, hyperacidity, Helicobacter pylori infection, stress, psychological disorders and abnormalities and genetic predisposition play a role in FD (8, 9). Moreover, there is increasing evidence for the involvement of the immune system in FD (10). 

Recent researches have indicated the importance of immunological mechanisms for the understanding of pathophysiology of FD. Differences in the individual cellular immune response may reflect the clinical diversity (5). The intestinal intraepithelial lymphocytes are likely to be important in the preservation of mucosal integrity and the vast majority of these cells are of T-cell type and more than 70% are CD4+ or CD8+ T-cells (11, 12). CD4 and CD8 T cells are the major part of T-lymphocytes. After activation and differentiation to distinct effectors’ subtypes CD4 T cells play a crucial role in mediating immune response through the secretion of specific cytokines (13).

Limited inflammatory processes in the gastric mucosa are caused by the influence of immune cells which result in functional dyspepsia (14). Using immunohistochemical techniques the majority of lymphocytes in the background were shown to be T cells with an increase in helper/suppressor CD4/CD8 ratio (15). FD is highly prevalent in the northwest of IRAN (16). The fact that very little is known about the immunopathology of the disease and its underlying mechanisms, we try to check for a possible immune mediated mechanism. In the current study, two groups of patients: functional dyspepsia with stomach pain and functional dyspepsia with abdominal bloating without gastric diseases such as peptic ulcer and gastric cancer were investigated. Our study was conducted to document the membrane expression of the CD4+ and CD8+ T-cell in the gastric mucosa of patients with FD and control group without H.pylori infection to provide arguments for an immunological process in FD.

## Methods

In this study, a total of 91 individuals, including 61 patients with FD (35 patients with stomach pain and 26 patients with abdominal bloating) and 30 healthy subjects admitted to endoscopy section at referral Imam Reza Hospital, Tabriz University of Medical Sciences/Iran were investigated for two years. Tabriz is one of the largest cities in Iran located in northwestern Iran (16).

**Patients and controls: **The diagnosis of FD was done according to Rome III criteria. A Rome III diagnostic criterion of FD requires one or more of the following symptoms: (1) bothersome postprandial fullness, (2) early satiation, (3) epigastric pain, and (4) epigastric burning .All controls were referred to endoscopy and eligibility criteria for control group were negative history of gastrointestinal diseases, normal physical exam, normal proximal endoscopy, normal abdominal and pelvic ultrasonography, and Helicobacter pylori-negative. It is to be noted that H.pylori were examined by histopathology method and h. pylori antigen stool test in the patient and control groups, respectively. The use of drugs in the last 2 weeks and the presence or absence of troublesome GI symptoms over the preceding 3 months were considered as exclusion criteria.

1. Bothersome postprandial fullness, (2) Early satiation, (3) Epigastric pain, and (4) Epigastric burning.

**Prepartion slides: **A gastric mucosal biopsy specimen was used for histopathologic evaluation by pathologist in both groups to determine the status of CD4 + and CD8 + T cells (presence and percentage of these cells) and CD4+/ CD8+ratio. 

The slides were prepared and stained as follows:

1. Preparation of 4 micron slices from paraffin blocks
2. Put at 37 ° C for one night for dehydration
3. Keep in citrate buffer at 120 ° C for 10 minutes
4. Wash with hydrogen peroxide solution 3% for 10 minutes
5. Dry the samples
6. Add primary antibodies of CD4 and CD8 for 10 minutes
7. Wash with phosphate-buffered saline
8. Add envision solution for 5 to 10 minutes
9. Wash with phosphate-buffered saline
10. Add chromogen solution and stain with hematoxylene
11. Hydration
12. Examination of the prepared slides using an electron microscope

**Data and statistical analysis: **A standardized questionnaire was used when visiting patients. The variables included age, gender, history of GI diseases, rapid urease test as negative or positive, and the results of physical exam, proximal endoscopy, abdominal and pelvic ultrasonography. Furthermore, the CD4 and CD8 T-cells were isolated from gastric mucosal biopsy from the antrum of each patient and quantified by microscopy. Immunohistochemical assay was used to detect the infiltration of CD4 and CD8 cells in frozen sections of the gastric mucosa. The CD4 and CD8 counting process was blinded to the case/control patients. Data were analyzed using SPSS 18 for frequency, percentage, mean, standard deviation, minimum, and maximum. The comparison between variables was analyzed with a chi-square or Fisher’s exact test. Logistic regression analyses were done to ascertain variables independently associated with cases and control. P<0.05 and odds ratio (OR) with a 95% confidence interval demonstrated statistical significance.

**Ethical standards: **This study was approved by the Tabriz University Ethics Committee No =24/17 and conducted in accordance with the revised Declaration of Helsinki. All participants gave their informed consent to participate in the study.

## Results

In this study, a total of 91 individuals, 61 patients with FD (35 with stomach pain and 26 with abdominal bloating) and 30 healthy subjects admitted to the endoscopy section at referral Imam Reza Hospital, Tabriz University of Medicine/Iran, were included in our analysis and were investigated for a 2-year period. Tabriz is one of the largest cities in Iran and located in the North-West Iran (17). Demographic data of this study is shown in table 1. 

**Table 1:** Demographic characteristics of patients and controls

**Variables**	**Cases** **N=61**		**Controls** **N=30 (33)**
	**35 (38.5)**	**26 (28.6)**	
Male	15 (42.9)	11 (42.3)	13 (43.3)
Female	20 (57.1)	15 (57.7)	17 (56.7)
Mean ± SD	47.62±1.22	44.72±1.25	45.63±1.17
Median (IQR)	48.5 (26-63)	42 (27-63)	47(26-63)
Symptom	Stomach pain	Abdominal bloating	Negative
History of GI Diseases	Negative	Negative	Negative
Physical examination	Normal	Normal	Normal
Proximal endoscopy	Normal	Normal	Normal
Abdominal and pelvic ultrasonography	Normal	Normal	Normal
Helicobacter pylori	Negative	Negative	Negative

SD: standard deviation;

IQR: minimum-maximum;

The numbers of mean and median are based on year and the rest based on frequency (percent).

All patients were H. pylori-negative and no patients had received antibiotics and/or immune-modulating drugs within the 4 weeks before endoscopy. The characteristics of CD4+ and CD8+ cells in the gastric mucosa of FD patients and controls are shown in table 2. 

**Table 2:** Pathological characteristics of patients and controls

**Variables**		**Cases N=61**		**Controls** **N=30 (33)**
		**35(38.5)**	**26(28.6)**	
CD4+ T cells	Positive	0 (0)	0 (0)	5 (16.7)
	Negative	35 (100)	26 (100)	25 (83.3)
CD8+ T cells	Positive	2 (5.7)	0 (0)	6 (20)
	Negative	33(94.3)	26 (100)	24 (80)
CD4+ T cells	Mean ± SD	-	-	4.73±16.94
	Median(IQR)	-	-	47 (26-63)
CD8+ T cells	Mean ± SD	4±17.17	-	8.4±24.48
	Median(IQR)	0 (0-90)	-	0 (0-90)

SD: standard deviation; IQR: Minimum-Maximum; The numbers of mean and median are based on year and the rest based on frequency (percent).

**Association between patients and controls based on the existence of CD4 and CD8: **For the investigation of association between disease and the presence of T cells, first we compared functional dyspepsia patients and healthy individuals based on CD4+ and CD8+ T-cell counts in gastric mucosa.

Second, patients were divided into two groups according to demographic symptoms, 35 patients with stomach pain and 26 with abdominal bloating and compared with 30 healthy individuals. T test and a logistic regression model were developed to examine the T cell presence between the 2 groups of patients and controls. Results of logistic regression analysis explained the adjusted OR for the final model. The results are shown in table 3. 

Results demonstrated that FD patients showed a decrease in mucosal immune cells compared to control groups with significant difference (P=0.003, P=0.008). CD4 was very rare in FD patients while it was detected in most of the controls. Patients were divided into two groups: patients with stomach pain and patients with severe bloating. Patients with stomach pain showed a decrease in CD4 count compared to the control group (P=0.01) and patients with severe bloating showed a decrease in CD8 count compared to the control group (P=0.002). 

Overall, CD4 and CD8 showed a significant difference between FD patients and controls. As there is a significant difference between patients and control regarding the presence of CD4 and CD8, it could be concluded that CD4 and CD8 have a protective role in functional dyspepsia.

**Table3:** Logistic regression analysis with the dependent variable in the final model

**Groups**	**Variables**	**P-Value**	**Adjusted OR**	**95% CI Adjusted OR**	
				**Lower**	**Upper**
Case/Control	CD4+ T cells (P/N)	0.003	-	-	-
	CD8+ T cells (P/N)	0.008	0.13	0.02	0.72
Case. Stomach Pain/Control	CD4+ T cells (P/N)	0.01	-	-	-
	CD8+ T cells (P/N)	0.08	-	-	-
Case. Abdominal bloating/Control	CD4+ T cells (P/N)	0.08	-	-	-
	CD8+ T cells (P/N)	0.002	-	-	-

P/N: Positive/Negative; OR: Odds Ratio; The numbers of mean and median are based on year and the rest based on frequency (percentage).

## Discussion

Dyspepsia is a common clinical condition associated with a complexity of the upper abdominal symptoms. Dyspepsia can have multiple causes, including gastroesophageal reflux disease, peptic ulcer or functional dyspepsia. Functional dyspepsia is a disorder characterized by upper-centered discomfort or pain, feeling of abdominal fullness, early satiety, abdominal distention and bloating, belching, and nausea. Briefly, FD is characterized by the presence of chronic or recurrent symptoms of upper abdominal pain or discomfort and absence of any known specific structural cause (18). The pathophysiology of FD is probably multi-factorial and not completely understood. The results of this study in patients with GI suggested that immune dysfunction may play a role in FD. Although several studies have been conducted on the probable role of humeral and cellular immunity, there were only a few published studies about the role of cellular immunity in gastrological functional diseases (18-20). Mucosal CD4+ and CD8+ T lymphocytes carry out different functions during immune reactions, partly as a result of the distinct patterns of lymphokines that they secrete upon stimulation. 

Although CD4+ T cells are a major component of the gastric cellular infiltration in H. pylori infection, they are believed to be fundamental to the initiation and maintenance of gastric inflammation (19). Although T cells synthesize and release cytokines that may lead to disturbed gastric physiology, recent data in laboratory animals suggest that CD4+ T cells may also have a protective role, by their ability to produce endorphin which contributes to decrease sensory fiber activation and pain perception. However, the assessment of the presence of CD4+ T cells is necessary to determine whether FD was dependent on infiltration by CD4+ T cells or not. In this study, we examined the immune CD4+ T cells. We hypothesized that CD4+ T-cells not infected with H. pylori would differ in FD patients compared to controls.

According to a study conducted by Nwokediuko et al. in Nigeria, FD is associated with a high degree of inflammation in the duodenal mucosa (20). Gargala et al. quantified the intraepithelial lymphocytes in the duodenal mucosa of patients with FD. In contrast, results of the study by Walker et al. demonstrated that eosinophil cells, but not intraepithelial lymphocytes, significantly increased in FD (21). The results of this study suggest that the absence of CD4+ and CD8+ T cells may be associated with increased risk of functional dyspepsia. However, further studies involving larger sample size or describing effective factors should be performed to validate the relationship between CD4+ and CD8+ T cells, interfering factors and increased risk of functional dyspepsia.

In conclusion we aimed to measure T-lymphocyte-type CD4+ and CD8+ as indicators of the immune system in patients with Helicobacter pylori-negative functional dyspepsia. The symptoms of FD have a positive correlation with the presence of CD4+ and CD8+ T cells in the gastric mucosa due to various factors, and these T cells might play an important role in inflammation, which might be helpful in finding a more specific therapy for FD.

## Abbreviation:

FD: Functional dyspepsia; HP: Helicobacter pylori; GI: Gastrointestinal.

## Funding:

no Funding
